# Myasthenia gravis: the future is here

**DOI:** 10.1172/JCI179742

**Published:** 2024-06-17

**Authors:** Henry J. Kaminski, Patricia Sikorski, S. Isabel Coronel, Linda L. Kusner

**Affiliations:** 1 Department of Neurology and Rehabilitation Medicine and; 2 Department of Pharmacology and Physiology, George Washington University, Washington, DC, USA.

## Abstract

Myasthenia gravis (MG) stands as a prototypical antibody-mediated autoimmune disease: it is dependent on T cells and characterized by the presence of autoantibodies targeting proteins located on the postsynaptic surface of skeletal muscle, known as the neuromuscular junction. Patients with MG exhibit a spectrum of weakness, ranging from limited ocular muscle involvement to life-threatening respiratory failure. Recent decades have witnessed substantial progress in understanding the underlying pathophysiology, leading to the delineation of distinct subcategories within MG, including MG linked to AChR or MuSK antibodies as well as age-based distinction, thymoma-associated, and immune checkpoint inhibitor–induced MG. This heightened understanding has paved the way for the development of more precise and targeted therapeutic interventions. Notably, the FDA has recently approved therapeutic inhibitors of complement and the IgG receptor FcRn, a testament to our improved comprehension of autoantibody effector mechanisms in MG. In this Review, we delve into the various subgroups of MG, stratified by age, autoantibody type, and histology of the thymus with neoplasms. Furthermore, we explore both current and potential emerging therapeutic strategies, shedding light on the evolving landscape of MG treatment.

## Introduction

Myasthenia gravis (MG) is one of the best-understood antibody-mediated autoimmune disorders. Autoimmune destruction of the neuromuscular junctions (NMJs) that transmit motor neuron impulses to muscle fibers causes weakness in voluntary muscles that varies widely in severity and scope among affected individuals. A surge in innovative therapeutics for MG has occurred as a result of enhanced comprehension of its immunopathogenesis, rapid progress in drug development, and financial incentives encouraging rare disease drug research (1). The field has been fortunate to have robust animal models since the 1970s to characterize antibody effector mechanisms and cellular pathology (2). Cell-based assays, and, more recently, functional in vitro NMJs generated from human stem cells, have provided valuable platforms for screening potential therapeutics (3–7). Breakthrough treatments have moved from preclinical assessment to clinical trials, ultimately culminating in FDA approvals for treatment of MG, and hold potential for application in numerous related conditions.

Over 15 years ago, Kaminski and colleagues speculated on future research discoveries and MG treatments in a Review for the *JCI* (8); some of their predictions have proven remarkably prescient. For instance, the expectation that complement inhibitor therapy would become a reality has materialized with FDA approval. Conversely, the development of antigen-specific therapies has not progressed. In this Review, we concentrate on contemporary understanding of MG’s pathophysiology and new therapeutics. For a comprehensive historical account of pivotal discoveries in the realm of MG, please see the insightful Review by Angela Vincent and colleagues (9).

## Clinical phenotype and diagnosis

The hallmark of MG is muscle fatigue with a degree of weakness that can fluctuate over minutes and vary in severity over weeks to months. Clinically, patients are categorized as having ocular myasthenia, which is characterized by complaints of ptosis or diplopia or both, and generalized MG, which involves weakness of any voluntary muscle. Generalized weakness can range from highly isolated manifestations, particularly bulbar muscles, to widespread muscle weakness, including respiratory insufficiency producing respiratory failure (10). Despite resolution of manifest weakness with treatment, many patients complain of general fatigue, as assessed by patient-reported outcome measures (11–13) and patient survey (14). This symptom suggests an etiology outside neuromuscular transmission compromise, which could be explained by concomitant sleep disturbance, psychological factors, and likely the pathological immune reaction, given the common observation of fatigue in other autoimmune disorders (15).

Once clinically suspected, serologic or electrodiagnostic testing can be used to confirm the diagnosis of MG (16, 17). Approximately 80% of patients with generalized MG and half of those with ocular myasthenia exhibit elevated levels of antibodies against the nicotinic acetylcholine receptor (AChR). Recently, cell-based assays have been developed with plasma membrane expression of the AChR, allowing the antigen to resemble the native receptor more closely (18). Muscle-specific kinase (MuSK) antibodies are found in 5%–8% of patients without AChR antibodies (19). Antibodies against lipoprotein receptor–related protein 4 (LRP-4) may be found associated with the MuSK or AChR antibodies or in isolation, although they are also present in patients with motor neuron disease and patients without evidence of diseases (20–22). Repetitive stimulation studies and single-fiber examination confirm the diagnosis in patients without positive serology in 90% of patients (16). Repetitive ocular vestibular-evoked potentials, magnetic resonance imaging of the extraocular muscles, and specialized neuro-ophthalmologic examinations have been evaluated to assist in diagnostic confirmation (23, 24).

## Neuromuscular transmission compromise in MG

**Figure 1:**
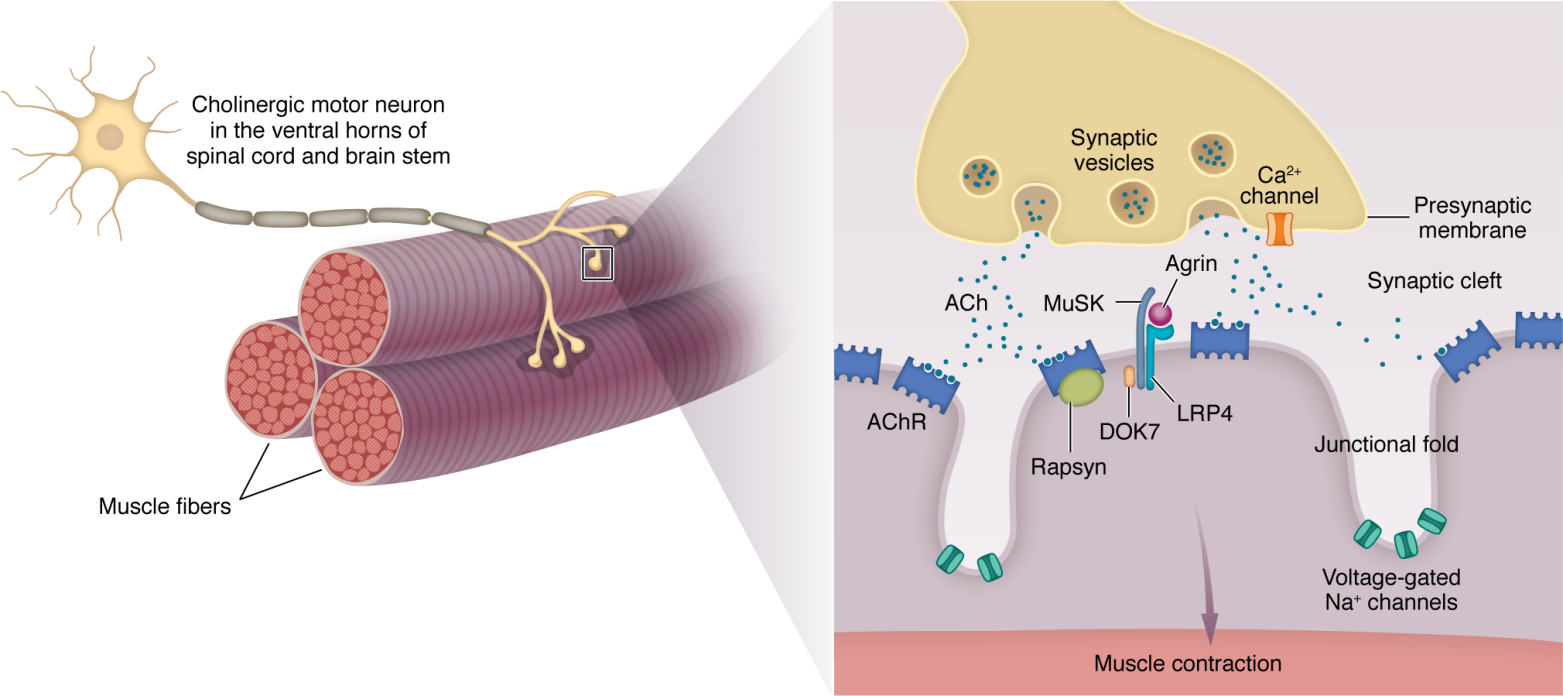
Structure of the NMJ. Each α–motor neuron axon divides into branches that innervate many individual muscle fibers. Each branch loses its myelin sheath and further subdivides into many presynaptic boutons, which face the surface of the postsynaptic surface of the muscle fiber and contain synaptic vesicles loaded with ACh. Between the synaptic bouton and the muscle surface lies the synaptic cleft, which contains acetylcholinesterase. The postsynaptic membrane has characteristic invaginations, with the AChRs densely packed at their tops. AChR density is influenced by both clustering and declustering signals, including ACh itself. Agrin, secreted by the nerve, binds to LRP-4 on the postsynaptic membrane, enhancing its binding with MuSK, which leads to MuSK autophosphorylation and ultimately the clustering of AChR. Rapsyn, a cytoplasmic protein, anchors AChR to the muscle cytoskeleton. When the nerve action potential reaches the synaptic bouton, voltage-gated Ca^2+^ channels are activated, leading to the fusion of synaptic vesicles with the nerve terminal membrane and release of ACh. ACh diffuses across the synaptic cleft, with some binding molecules the AChR. Binding triggers AChR ion channel opening, permitting influx of Na^+^ into the postsynaptic region. The resulting EPP activates voltage-gated Na^+^ channels at the bottom of the folds, leading to further Na^+^ influx and spreading of the action potential along the muscle fiber. Other proteins, including Rapsyn, MuSK, Dok-7, LRP-4, and agrin, which are involved in AChR clustering, are also present on the muscle membrane in close proximity to the AChR.

The clinical phenotype of MG is driven by destruction of the NMJs, leading to impaired neurotransmission between motor neurons and muscle fibers. The components of the NMJ involved in neuromuscular transmission include the nerve terminal, synaptic cleft, and postsynaptic muscle surface, which are highly specialized to ensure dependable signal transmission (Figure 1) (25, 26). Neuromuscular transmission failure occurs owing to a reduction in the number or activity of AChR molecules at the NMJ, leading to a decrease in the end-plate potential (EPP). At rest, this EPP reduction may still adequately support neuromuscular transmission; however, during repetitive activity, when the quantal release of acetylcholine (ACh) is reduced, the EPP may fall below the threshold required to trigger an action potential. Neuromuscular fatigue, characterized by a progressive loss of force generation, occurs as increasing numbers of muscle fibers become incapable of contracting. This phenomenon explains the clinical hallmark of MG of fatiguing muscle weakness.

## Effector mechanisms of autoantibodies

Individuals with MG produce autoantibodies that mediate disruption of the NMJ leading to compromised neuromuscular transmission. Below, we discuss the mechanisms of NMJ injury associated with the MG-associated antibodies AChR and MuSK as well as other autoantibodies identified in patients with MG.

### AChR antibodies.

**Figure 2:**
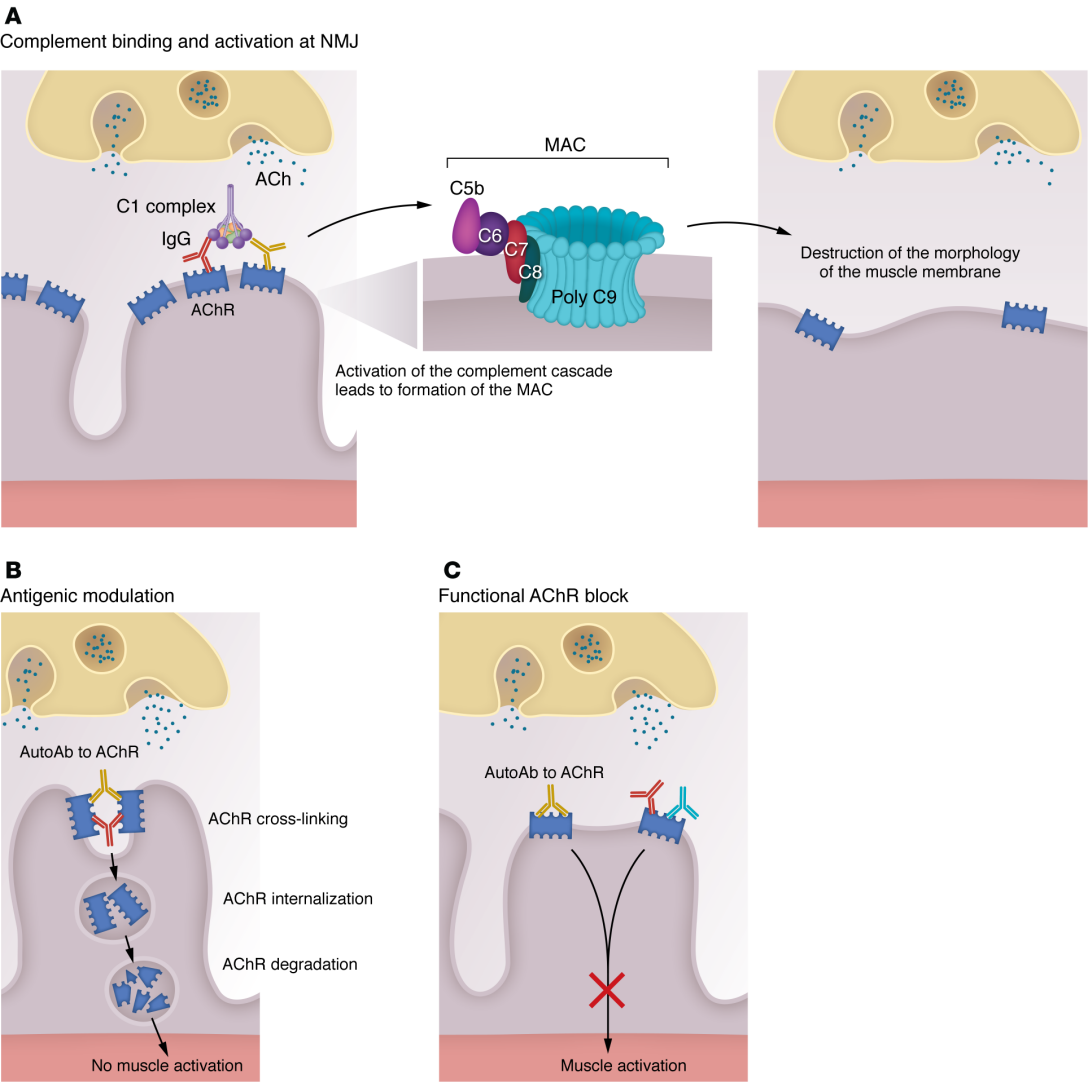
Effector mechanisms of AChR antibodies. (**A**) Antibody binding to the AChR activates the complement cascade, resulting in the formation of membrane attack complex (MAC) and localized destruction of the postsynaptic NMJ membrane. This ultimately leads to a simplified, altered morphology of the postsynaptic membrane of the NMJ of patients with MG and EAMG animals. (**B**) Antibodies cross-link AChR molecules on the NMJ postsynaptic membrane, causing endocytosis of the cross-linked AChR molecules and their degradation (antigenic modulation). It is likely that antibodies attaching to different epitopes are required to produce modulation and complement activation. This ultimately leads to a reduced number of AChR molecules on the postsynaptic membrane. (**C**) Antibody binding of the ACh-binding sites of the AChR causes functional block of the AChR by interfering with binding of ACh released at the NMJ. It is important to appreciate that there may be overlap in the pathogenic mechanisms of individual AChR antibodies and these mechanisms may cooperate to induce disease.

AChR antibodies impair neuromuscular transmission through three mechanisms, as summarized in Figure 2 and described below. While these mechanisms are well established, the precise contribution to weakness in individual patients varies and can change over the course of the disease and be influenced by treatment. Additionally, individual antibodies can induce disease through multiple mechanisms, and their cooperation may be necessary to produce injury (7, 27). Interestingly, some patients exhibit elevated AChR antibody levels without clinical evidence of disease. In such cases, AChR antibody cannot be pathogenic (28). This observation might be due to antibodies targeting epitopes that are hidden from attack, such as the cytoplasmic portions of the AChR, or they may be incapable of inducing injury through any of the three described mechanisms (29).

### Complement activation.

Evidence in both humans and animal models (experimental autoimmune MG [EAMG]) has demonstrated the importance of complement activation in mediating disease in patients with AChR antibody-positive MG (30). Upon binding of AChR antibody, the membrane attack complex is formed on the postsynaptic surface and leads to the shedding of AChR-rich membrane resulting in the loss of synaptic folding as well as Na^+^ channels (Figure 2A). The inhibition of the complement component C5 activation prevents membrane attack complex formation (31), and this has translated to FDA approval of three drugs (32–34). Importantly, some patients do not respond well to complement inhibition, underscoring the relevance of other mechanisms involved in disease induction. Furthermore, an inadequate level of complement inhibition could still produce injury at the critical site of pathology, the NMJ (35).

### Antigenic modulation.

This refers to the capacity of an antibody against link two antigen molecules, instigating a cellular signal that expedites the process of endocytosis and subsequent degradation of the complex (Figure 2B) (7, 27, 36–38). Cooperation among antibodies binding different antigens on the AChR is likely required for modulation as well as effective complement activation. The necessity for cooperation among antibodies to trigger pathogenesis could further explain why circulating AChR antibody level does not correlate well with clinical disease.

### Blockade of AChR function.

This occurs when antibodies bind to the ACh binding site (Figure 2C). In some patients with MG, there may be trace amounts of AChR antibodies that specifically recognize the cholinergic site. Such antibodies are expected to have a rapid and severe effect on neuromuscular transmission (39). Functional assays using in vitro human NMJ models support the hypothesis that AChR antibodies will induce a blockade neuromuscular transmission independent of other mechanisms (3, 40).

### MuSK antibodies.

MuSK antibodies interfere with clustering of AChR on the postsynaptic muscle surface directly opposed to the nerve terminal. Antibodies against MuSK are primarily IgG4, which do not have covalently bound Fab arms, in contrast to other IgGs (41, 42). IgG4 antibodies in circulation undergo continuous exchange, becoming functionally monovalent, and the monovalent MuSK antibody compromises clustering and induces disease. Patients with MuSK MG have low levels of other IgGs, which can reduce clustering or activate complement, but the degree to which these are pathogenic in humans has not been studied extensively (43).

Reported prevalence of MuSK MG shows regional variability (44–47). A relative preponderance of patients with MuSK MG are women, with an age of onset peaking in the third to fourth decade (48, 49). Patients with MuSK antibodies demonstrate a propensity for involvement of bulbar muscles, and experimental models support greater disruption of NMJ architecture by MuSK antibodies, supporting fundamental differences among muscles in mechanisms of synapse maintenance (50). In addition, patients with MuSK antibodies tend to respond poorly to cholinesterase inhibitors. This could be explained by the increase in ACh providing a de-clustering signal (26).

### Seronegative myasthenia.

Upward of 10 percent of patients may not have detectable circulating autoantibody against AChR or MuSK. These patients fall into two major groups: those who have AChR antibodies, which may be detected by cell-based assays (51), and those who have antibodies directed toward other NMJ proteins (4, 52). In animals, LRP4 was found to be a targeted antigen in seronegative MG, with a low prevalence of 1%–5% of the MG population (4, 52, 53). LRP4 functions as a critical protein at the NMJ by binding agrin and initiating AChR clustering with the assistance of MuSK (54–56). The LRP4 antibodies are primarily IgG1 and IgG2 subtypes and follow similar clinical presentation to a mild form of early-onset MG (22), inhibit clustering of AChR, and appear not to strongly activate complement (52, 57). Interestingly, LRP4 antibodies have been found in patients with AChR and MuSK antibodies with greater levels of disease severity (22).

Patients without these other autoantibodies are a highly heterogenous group, about which there is limited information regarding disease mechanisms. Other antigen targets have been suggested as the potential binding sites for antibodies that cause weakness, such as agrin, titin, Kv1.4, ryanodine receptor, collagen Q, and cortactin (4, 58). These antibodies likely do not contribute to weakness, but rather reflect a more general deterioration in tolerance, as observed in late-onset and thymoma-associated MG. Antibodies against agrin are seen in seronegative patients and are coexistent with AChR antibodies, but their pathogenicity has been studied in a limited fashion (59, 60).

## Cellular pathogenesis of MG

**Figure 3:**
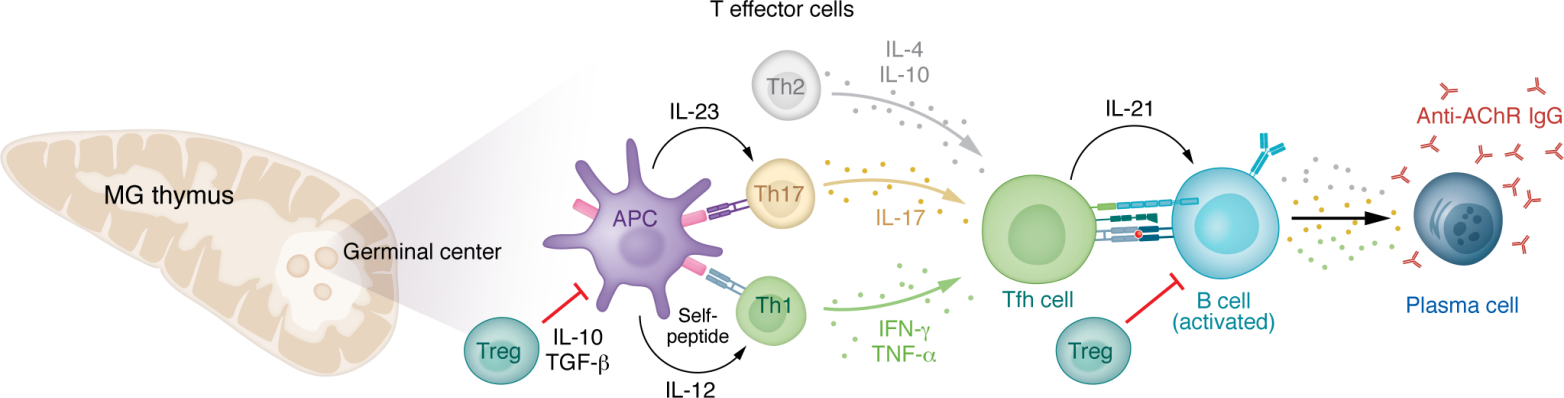
Cytokine network and cells involved in the pathogenesis and immunoregulation of AChR antibody MG. Th1 cytokines stimulate production of IgG subclasses that bind and activate complement effectively, whereas Th2 cytokines stimulate the production of Ig classes and IgG subclasses that do not. See text for details. AChRs are presented to naive T cells via antigen-presenting cells (APCs), leading to production of IL-23 and IL-17 that contributes to tissue inflammation in the MG thymus. Increased levels of Th1 cytokines (IFN-γ) promote the T follicular helper (Tfh) cell interaction with the recruited B cells. Th17 proinflammatory cytokine levels (IL-17) promote differentiation of B cells into antibody-secreting cells and production of complement-fixing antibodies. Tfh cells secrete IL-21, which promotes plasma cell differentiation. Tregs modulate proinflammatory responses by secreting antiinflammatory cytokines to suppress T cell and B cell responses. Dysfunction in circulating and thymic Tregs is associated with MG pathogenesis.

**Figure 4:**
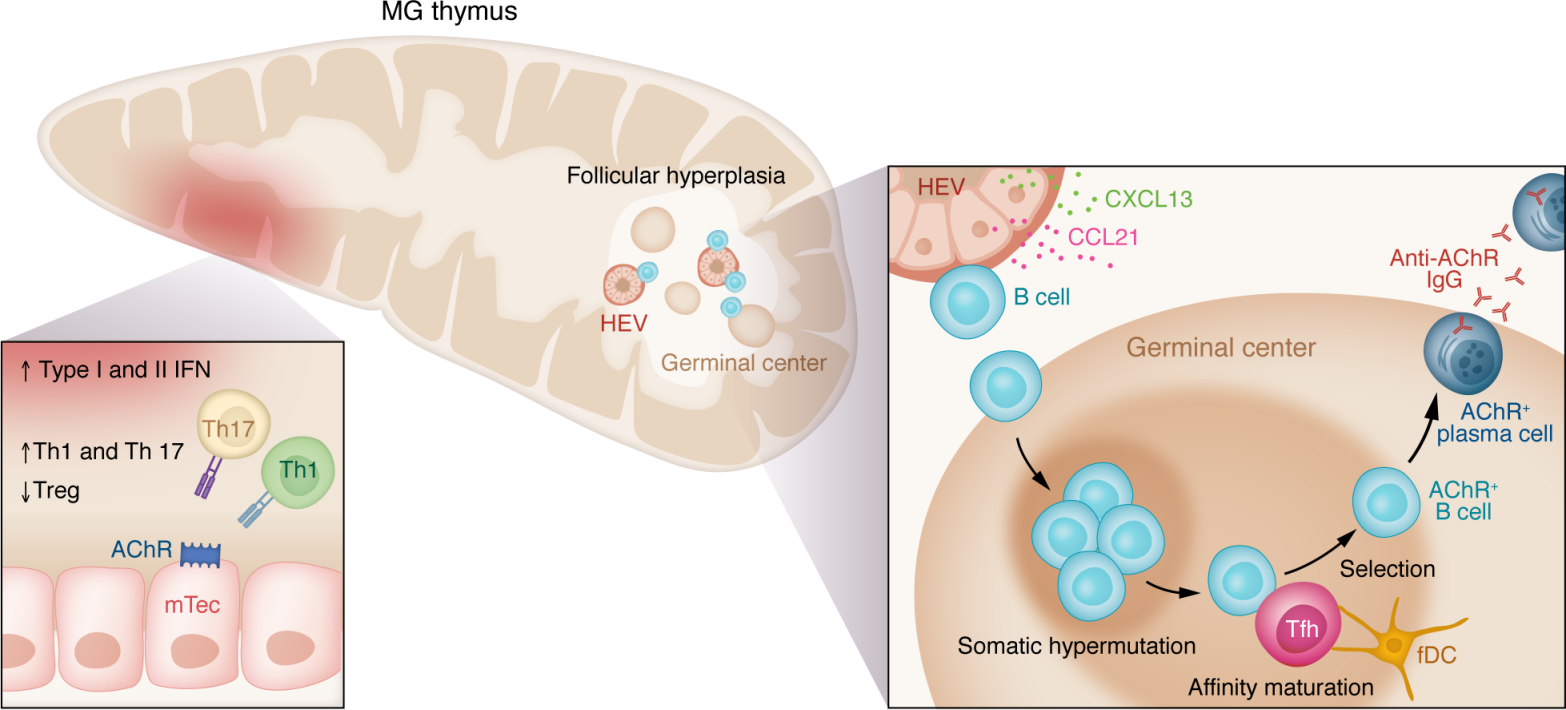
Thymic pathology associated early-onset MG. The thymus is the organ of T cell maturation and establishment of central tolerance. Self-peptides are presented by medullary thymic epithelial cells (mTECs). Self-reactive T cells undergo apoptosis or are controlled by Tregs; however, suppressor functions of thymic Tregs are impaired in MG. Type I and II IFN induction in the thymus promotes expression of AChR, cytokines, and chemokines by thymic epithelial cells. Increased expression of IL-17 and IL-23 promotes expansion of Th1/Th17 cells. High endothelial venules (HEVs) and secretion of CCL21 and CXCL13 facilitate recruitment of B cells and ectopic germinal center formation associated with thymic hyperplasia. In the germinal center, B cells undergo somatic hypermutation, affinity maturation, and selection, processes that are implicated in development of AChR^+^ long-lived plasma cells. Anti-AChR–producing plasma cells exit the germinal center and migrate to the bone marrow. fDC, follicular DC.

Substantial advancements in understanding MG pathophysiology have led to growing recognition of various mechanisms contributing to disease development in different patient groups. The clinical categories of ocular and generalized MG can be divided further based on autoantibody status, age, and thymic pathology (Table 1). Those who test positive for AChR antibodies are subdivided into early-onset and late-onset groups, typically distinguished by onset before or after 45–50 years of age (61). Uniformly, MG is an antibody-mediated disease, with B cells requiring T cell help; increasing appreciation of fundamental dysregulation of T cell function leading to compromised immune checkpoints highlights autoreactive B cells as a driver of pathology (Figure 3) (62–64). Cytokine signals support MG pathology (Figure 4). The pathophysiology of each of the subgroups is elaborated upon below.

### Early-onset myasthenia.

The best-characterized form of MG is in patients younger than 50 years, as investigators have taken advantage of evaluation of the pathological thymus after its surgical removal. Transcriptional profiling of RNA and miRNA has found a proinflammatory signature (65, 66) with elevations of various cytokines, including IFN-β, IL-17, IFN-II, TGF-β, and others, which support autoreactive B cell development (Figure 3) (67). Patients with early- and late-onset MG have elevated miRNA-150-5p and miRNA-21-5p in serum. Both miRNAs are decreased with immunotherapy, and thymectomy reduces levels of miRNA-150-5p in circulation (68). The thymus of early-onset patients demonstrates follicular hyperplasia manifesting as an increase in lymphoid follicles and perivascular spaces (Figure 4) (69). B cell infiltration and germinal center formation is associated with overexpression of CXCL13, CCL21, and B cell–activating factor (BAFF) in thymic epithelial cells (70, 71). Increased numbers of high endothelial venules around germinal centers in the hyperplastic thymus indicate active trafficking of lymphocytes (70, 72). IFN-β, a cytokine signature associated with the MG thymus, has been shown to induce the production of these chemokines by thymic epithelial cells in vitro (70).

While the thymic cortex appears normal in early-onset MG, the medullary areas are increased in size with lymphoid follicles and diffuse B cell infiltrates with AChR antibody–producing cells (Figure 3). The MG thymus also has muscle-like cells and thymic epithelial cells that express AChR-like proteins. A deficiency of intrinsic complement regulatory proteins is appreciated in these cells, with evidence of complement protein deposition on their cell surface. These observations have led to the hypothesis that ongoing complement attack of myoid and epithelial cells promotes germinal center formation (73). Some have hypothesized that a deficiency of macrophages leads to impaired removal of necrotic thymomcytes, promoting the proinflammatory environment (74). This would lead to activation of other self-reactive CD4^+^ cells by antigen-presenting cells with epitopes derived from the injured tissue, causing further tissue destruction and sensitization of CD4^+^ cells to an increasingly larger repertoire of tissue epitopes and antigens (“epitope spreading”) (75). Thymocytes produce AChR antibodies, as do circulating plasma cells (76). All these observations support the pathological thymus as the originating site of autoreactivity in early-onset MG, but once initiated, the autoimmune process remains active despite removal of the thymus. Circulating AChR antibody, thymus-derived B cells (77), and autoreactive T cells remain, with many patients continuing to show clinical signs of disease (78).

Much like other autoimmune conditions, a genetic predisposition contributes to development, with twin and family studies consistently demonstrating elevated prevalence rates of MG and other autoimmune disorders (79, 80). Association of HLA A1-B8 and DR3-DQ2 (AH8.1), which is shared with many autoimmune diseases, has been appreciated for decades for early-onset MG in White populations (81, 82). Strong associations for early-onset MG are appreciated for SNPs in *S100P*, *GAB2*, *NFKBIA*, *TNFAIP3*, and *PPP1R15A* genes. Pathway analysis combining several GWAS support a particular signature of genes associated with the innate immune system and therefore supporting mechanisms common to response to viral infections (83).

Viral infection has been posited for decades to be a contributor to MG, with thus far limited support. Contradictory studies exist for the presence of Epstein-Barr virus existence in the MG thymus (84, 85). No evidence suggests other infectious agents to be associated with MG (86). Release of double-stranded DNA from necrotic macrophages may trigger the inflammatory and subsequent autoimmune reaction in the hyperplastic thymus (74). Expression profiling of the thymus supports dysregulated apoptotic pathways (65). A global propensity for increased cell death does not occur in MG thymus, as the antiapoptotic protein is increased (65).

### Late-onset MG.

The frequency of autoimmune disorders increases with age (87), and therefore, late-onset MG pathogenesis will likely share mechanisms with other later-onset autoimmune disorders (88). Late-onset MG affects men at a slightly higher frequency than women and is characterized by thymic atrophy and the presence of antibodies against titin and ryanodine receptor. Based on GWAS investigations, a clear genetic distinction is appreciated among patients above or below the age of 45–50 years, with consistent identification of SNPs associated with *CTLA-4*, *PTPN22*, and *TNFRSF11* (61, 83, 89). Each of these genes plays a role in T cell tolerance. Additional analysis focused on *CTLA-4* supported that polymorphisms in regulatory regions of *CTLA-4* would reduce the expression level of this critical immune checkpoint. A polymorphism in the regulatory region of the α subunit of the AChR was also found (61). Altered expression of the autoantigen AChR suggests two mechanisms for disease susceptibility: (a) aberrant AChR expression promotes a breakdown in tolerance in concert with age-related enhanced autoimmunity or (b) altered expression on the postsynaptic surface could make patients more susceptible to compromised neuromuscular transmission as they age.

Late-onset MG occurs in the context of immune system aging, which is associated with reduced ability to fight infections, reduced response to immunization, increased risk of cancer, and increased rates of autoimmunity (87, 90). Normal or accelerated biological aging in patients may combine with other factors to stimulate MG. From the first year of life the thymus begins to involute, with significant atrophy having developed by the age cut-off for late-onset MG, and the thymi of normal elderly and late-onset MG show no definitive differences; however, this does not eliminate the potential for functional differences. In the MGTX trial (which included thymectomy in both early- and late-onset MG), there were no differences in patient thymi across age groups with regards to germinal center counts, and treatment response did not correlate with thymic hyperplasia (68, 91).

### Thymoma-associated MG.

Approximately 10% of patients with MG, nearly all of whom have AChR antibodies, have a paraneoplastic form of the disease triggered by the thymoma. About one-half to two-thirds of patients with thymoma have an autoimmune disease, predominantly MG, which may occur as the presenting symptom of the tumor or may develop after resection. Thymomas differ in cellular composition, and those with predominance of immature lymphocytic components are most likely to be associated with autoimmune diseases (92). The autoimmune regulatory protein AIRE, which is responsible for expression of self-epitopes to allow for negative selection of autoreactive T cells, is absent in close to all thymomas irrespective of the presence of MG (78). Thymomas from patients with MG express fragments of muscle protein epitopes expressed in a subset of medullary thymic epithelial cells (93), which accounts for the expression of autoantibodies against not only AChR, but also titin and the ryanodine receptor as well as neurofilament protein (93). Development of MG requires the generation of autoreactive T cells within the tumor and subsequent exit of these T cells to the periphery and is further associated with a reduction of Tregs (94, 95). Gain-of-function mutations in CTLA4 and PTNP22 are observed in thymoma-associated MG (96, 97), likely leading to a loss of negative selection and suggesting common pathophysiological pathways with late-onset MG. With egress of autoreactive T cells, MG would be maintained by mechanisms independent of the tumor. Even with tumor resection, a threshold of pathogenic AChR antibody production would need to occur to manifest clinically. Such a scenario is consistent with patients developing MG years after tumor removal and the presence of AChR antibody in the sera of patients without manifest disease. A single-cell sequencing study of MG thymoma demonstrated all required cells and signaling molecules to promote autoreactive B cell formation (92, 93). Transcriptional profiling studies have identified divergent mechanisms of MG development based on the type of thymoma (98).

### Immune checkpoint inhibitor MG.

The development of immune checkpoint inhibitors (ICIs) has led to dramatic benefits for patients with treatment-resistant malignancy, but the unrestrained activation of the immune system has led to de novo induction or worsening of autoimmune disorders in at least 20% of patients depending on the agent and neoplasm (99, 100). ICI MG also differs markedly from other forms of MG (Table 1). Inhibitors of CTLA-4 and PD-1 induce MG, at times in apparent isolation but frequently in association with myositis, which also occurs in thymoma-associated MG but is otherwise not seen in early- or late-onset MG. The most extensive evaluation of ICI therapy–producing MG to date was a retrospective evaluation of 65 patients, a subgroup comprising less than one-quarter of patients treated with ICIs (101). The median age of the subgroup was 73 years, with two-thirds being men. At least one-third of patients in the subgroup had coincident myositis. Two-thirds of patients had elevated AChR and striated muscle antibodies (101). In toto, the data support fundamental clinical and pathophysiological differences in ICI-related MG compared with other forms of MG.

Anti–CTLA-4 drugs likely activate existing self-reactive T cell clones with concomitant suppression of T regulatory responses and stimulation of B cell response (102). Humans normally harbor self-reactive T cells (103), and therefore, presumably CTLA-4 inhibition activates existing autoreactive T cells, which drive development of autoantibodies. This mechanism would explain why ICI MG can occur in combination with myositis with expression of antibodies against multiple epitopes. However, some patients with clinical and electrophysiological evidence of MG do not have antibodies, which suggests similarities to seronegative MG. In contrast to CTLA-4–targeted drugs, PD-1 inhibitors cause expansion of T cell clones within the neoplasm, and therefore, autoreactive T cells may develop as part of the immune attack on tumor antigens.

### Ocular myasthenia.

This subgroup is clinically defined by manifestations restricted to the ocular muscles for its entire course (104). About one-half of patients have no detectable autoantibodies, by conventional assays, but performing cell-based assays, including those with a mix of fetal and adult AChR isoforms, increases their identification (105, 106). Ocular myasthenia with MuSK antibodies or thymoma is extremely uncommon. CD4^+^ T cells from individual patients with ocular myasthenia rarely recognize all the AChR subunits, even among patients with a long duration of disease, suggesting limited pathophysiological progression (107). Thymectomy, when performed, has identified thymic hyperplasia as well as atrophy (108). Higher rates of ocular myasthenia are appreciated in older American and Japanese populations (109, 110), while a study in China found higher rates of ocular myasthenia in children (111), with differential susceptibility based on HLA-DQA1/DQB1 haplotypes (112). Environmental factors were suggested based on latitudinal variation in ocular myasthenia frequency (113). The miRNA 30-e-5P is a potential biomarker to predict generalization of ocular MG to widespread MG (114), but how it relates to disease pathology is not known.

### MuSK myasthenia.

Several lines of evidence demonstrate that MuSK MG is a distinct disease from AChR MG (9). The observation of predominant IgG4 antibodies in patients with MuSK MG is indicative of immune response that corresponds with the IgG4 autoimmune diseases (9) and the antiinflammatory properties of the IgG4 that include the inability to activate complement. Moreover, at the NMJ, MuSK antibodies create a pathophysiological disruption of synaptic clustering compared with destruction of the synapse by complement with AChR antibodies (115). The thymus is normal (116), and thymectomy does not benefit patients with MuSK with MG (117). HLA-DRB1*14 and HLA-DQB1*05 are associated with MuSK MG, and there is a limited set of TCR VJ rearrangements (9). Finally, the response of MuSK MG versus AChR MG to CD20 depletion (118) strongly suggests that the circulating lymphocytes differ, with MuSK antibody production requiring the differentiation of B cells into plasmablasts versus AChR antibody secretion by long-lived plasma cells (119). The immunological environments that create these antibodies remain unclear.

### Other autoantigens.

Observations of thymus changes in patients with early-onset MG, such as hyperplastic medullary epithelial cells, germinal centers, and complement deposition, are also appreciated in thymus of some seronegative patients (73).

Generally, seronegative patients have had variable thymus pathology, but one study did suggest an inflammatory signature of the thymus in seronegative patients (73).

## Treatment

### Surgical therapy.

Thymectomy is a well-established treatment for patients with early-onset AChR antibody–positive MG (91), with sustained benefit up to 5 years after thymectomy. However, up to one-quarter of patients respond poorly and continue to require doses of prednisone and immunosuppressives. A recent investigation suggested that removal of the thymus in adults may lead to increased risk of neoplasia and autoimmunity (120). However, there is considerable disagreement with methods and conclusions of this study (121).

The precise mechanism by which thymus removal imparts clinical benefit is not fully understood. One likely hypothesis is that thymectomy eliminates a considerable source of antigenic stimulation, ultimately reducing the production of AChR antibodies, but autoreactive cells have exited the thymus remain and drive production of pathogenic antibodies (77).

### Pharmacological therapy.

Contemporary MG therapy encompasses a spectrum of medications that range from century-old treatments to cutting-edge first-in-human agents (Table 2). Comprehensive treatment guidelines have been established by national organizations and an international consortium (122–124), which the reader can review. Choice of treatment is influenced by several factors, including severity of disease, the patient’s individual characteristics, and the presence of comorbid conditions. These factors determine the tolerability of specific therapeutic agents and whether they align with insurance coverage or governmental regulatory approvals. As understanding of MG’s pathophysiology and treatment responses continues to evolve, there is increasing opportunity for personalized care plans that tailor treatment strategies to individual patient needs, optimizing the management of this complex autoimmune neuromuscular disorder. Furthermore, although prednisone is still the most consistently effective drug for MG, its side effect burden and that of immunosuppressive as well as the poor response in a large minority (125–127) has motivated development of new therapies. The sections below focus on treatments developed in the last decade and those under development.

### FcRn inhibition.

Endothelial cell surfaces express the neonatal Fc receptor (FcRn), which plays a crucial role in IgG antibody recycling. Antibodies in circulation bind to FcRn and are internalized, ultimately entering lysosomes, but are normally recycled back into circulation. FcRn inhibitors disrupt this binding within the lysosome, leading to the proteolytic removal of antibodies generally and including the subset of disease-causing antibodies. This results in significant reductions in circulating antibodies within days of the initial treatment. Efgartigimod and rozanolixizumab are approved for AChR antibody–positive MG (128, 129), with the latter further approved for MuSK antibody–positive MG. Thus far, these agents are exclusively for MG, but are likely to be used soon in other autoantibody-mediated conditions (130, 131). Trials of efgartigimod and rozanolixizumab in MG reported a subset of patients who responded poorly despite a reduction in circulating antibodies. Potential explanations are that the drop of circulating antibodies was not adequate for inducing a response or that remaining tissue-bound antibodies may maintain a significant level of disease. An alternative hypothesis that irreversible injury to the postsynaptic surface of the NMJ has occurred is unlikely, because (a) animal models demonstrate return of normal strength and neuromuscular transmission (132, 133), and, in some cases, ultrastructure of the NMJ begins to return to normal, and (b) patients with MG typically regain normal strength, even after experiencing a myasthenic crisis. Nipocalimab and batoclimab are FcRn inhibitors in clinical trials that differ in dosing regimens and may reduce circulating IgG to a greater extent than other agents (134).

### Complement inhibitors.

Another important advancement in MG therapeutics involves the inhibition of complement activation. To date, all FDA-approved drugs in this category focus on targeting the C5 convertase enzyme. Eculizumab is a humanized chimeric monoclonal antibody (135) designed to block cleavage of C5, thereby reducing the formation of the membrane attack complex by C5b and mitigating the proinflammatory effects of C5a. It is important to note that in MG there is no evidence suggesting a role for C5a in the disease.

Zilucoplan is a small macrocyclic peptide that not only binds to C5 to inhibit its cleavage, but also interferes with C5b binding to C6. This dual action theoretically provides a more effective means of limiting the formation of the membrane attack complex (136). Both eculizumab and zilucoplan have demonstrated efficacy in phase III trials (33, 34) and have received FDA approval. Ravulizumab, a modification of eculizumab with an extended half-life, is also in the clinic (32). There remain upward of thirty percent of patients who do not benefit from complement inhibition, which demonstrates the importance of other mechanisms of autoantibody action. An important concern with all complement inhibitors is enhanced risk of meningococcal and encapsulated bacterial infections (137).

Ongoing research efforts continue to explore the complement system as a target for MG treatment. For example, a siRNA that inhibits hepatic synthesis of C5 has shown promise in reducing the severity of EAMG (138, 139). Additionally, C7 has been effectively inhibited in EAMG models, further highlighting the potential of complement system targeting in MG therapeutics (30).

### B cell targeting.

B cell targeting is a notable area of therapeutic development for MG. A phase II study of rituximab, a chimeric antibody directed toward CD20 on B cells, in treatment-resistant MG did not achieve its primary outcome, while a phase III trial using rituximab within one year of disease onset improved clinical status (140). Presumably, patients with a shorter disease duration have pathogenic antibody produced by short-lived, CD20-expressing cells, in contrast with treatment-resistant patients. For MuSK MG, a blinded, prospective multicenter study demonstrated improved clinical status and reduced need for other immunotherapies (118). These investigations, along with deep cellular characterization of pathogenic B cells, suggest that MuSK MG is a disease of short-lived plasma cells (119).

CD38 is expressed on plasma, NK, and T cells and was targeted by TAK-079 in a phase II trial that showed promising safety results (NCT04159805). Other B cell monoclonal antibodies have been reported in single cases, but there are no ongoing trials involving them (141). Cladribine is a synthetic chlorinated deoxyadenosine that primarily inhibits B cell replication but also affects T cells; a pilot study of cladribine suggested efficacy (142), and a phase III trial is planned. Telitacicept is a fusion protein designed to inhibit B lymphocyte stimulator (BLyS) and proliferation-inducing ligand (APRIL), leading to suppression of development and survival of late-stage B cells and plasma cells (143). The drug is under phase III evaluation.

Proteasome inhibitors eliminate cells with high rates of protein production and are effective in treatment of multiple myeloma. In theory, these would be effective to eradicate antibody-producing cells of MG (144). An open-label trial of the proteasome inhibitor bortezomib was attempted but failed in recruitment (145).

CAR T cell–based therapies are being applied with B cell targets. An early-phase trial utilizing autologous RNA CAR T cell therapy against B cell maturation factor (BCMA), which is found on plasma cells, demonstrated efficacy and improved clinical outcome measures and is in phase II evaluation. An antigen-specific approach has been developed with engineered T cells to express a MuSK chimeric antibody receptor along with CD137-CD37, which would selectively eliminate B cells producing MuSK antibodies. The approach is in a phase I dose-finding evaluation. However, the superiority of CAR T cell–based approaches over existing treatments and their scalability for broader patient access remain open questions (146).

### Stem cell therapies.

Autologous human stem cell treatments have been restricted to single reports or case series restricted to treatment-resistant patients.

### Reestablishment of tolerance.

The loss of tolerance to NMJ proteins underlies MG, and attempts with some success have been made to reestablish tolerance for decades in animal models by administration of oral, nasal, or subcutaneous or synthetic AChR (147–149). A pilot investigation (NCT02609022) has been completed of an AChR peptide mimic with report of a good safety profile, which is critical, as administration of self-antigen could activate MG.

### Interference with cell signaling.

Belimumab is humanized immunoglobulin G1λ antibody that binds and blocks the activity of BAFF. This cytokine supports various aspects of development and maintenance of B cells, and its levels are elevated in patients with early-onset MG. Furthermore, polymorphisms in the *BAFF* gene enhance susceptibility to MG (150). Despite this favorable background and use in systemic lupus, a phase II study of belimumab failed to demonstrate efficacy (151). Iscalimab, an anti-CD40 antibody (152), blocks primary and recall T cell–dependent antibody responses and reduces germinal cell formation. A phase II study including AChR or MuSK antibodies in patients demonstrated safety but no to limited difference in clinical outcomes compared with placebo (152). Satralizumab, an inhibitor of IL-6, is undergoing a phase III trial for MuSK and AChR antibody–positive MG (NCT04963270).

## Conclusions and unmet needs

The definition of MG subtypes has led to the recognition that MG is not a singular disease but rather should be classified as an autoimmune disorder affecting postsynaptic transmission. This evolving understanding supports the likelihood that the next decade will offer opportunities for more precise therapy tailored to specific patient subgroups, as deeper insight into the distinct pathophysiological mechanisms become evident. As this knowledge expands, discovery of biomarkers that can predict treatment responses can be anticipated, which would have the potential to tailor therapy to optimize treatment selection and reduce adverse effects related to nonoptimal treatment choices. Studies of genetic polymorphisms and metabolomics have identified treatment-predictive markers (153–155), but further validation is necessary. There is a pressing need for such advancements, especially considering the proliferation of multiple costly treatment options with substantial minorities of patients showing a poor response. Furthermore, these treatments have been approved based on phase III placebo-controlled trials involving participants with highly selective inclusion and exclusion criteria, conducted over relatively short periods. While consensus guidelines are beneficial, they still rely on limited information. Additionally, individual clinicians may deviate from these guidelines owing to their relative inexperience with this rare disease and the financial or insurance-related constraints they encounter. Despite these challenges, the authors remain optimistic about the exciting prospects for further research in the field of MG.

## Figures and Tables

**Table 2:**
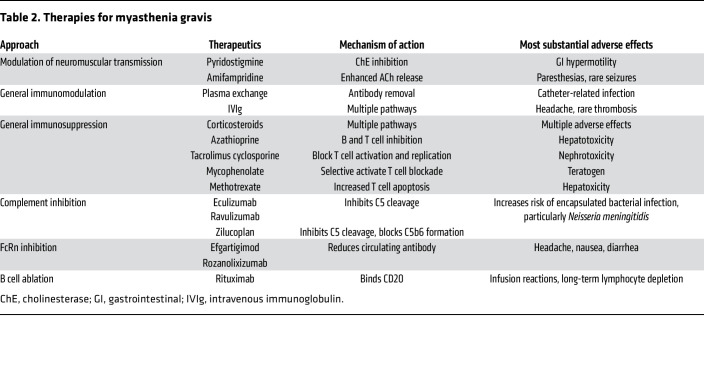
Therapies for myasthenia gravis

**Table 1:**
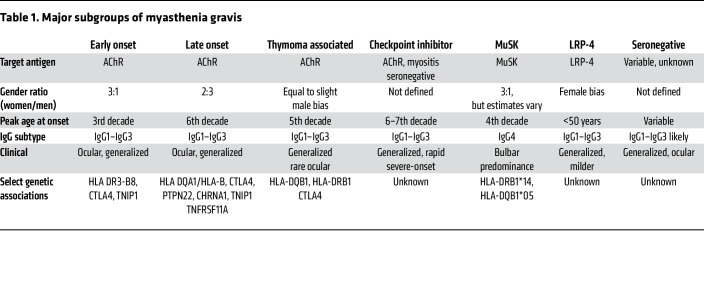
Major subgroups of myasthenia gravis

## Version 1 06/17/2024

Electronic publication

## References

[1] Nguyen-Cao TM (2019). Myasthenia gravis: Historical achievements and the “golden age” of clinical trials. J Neurol Sci.

[2] Kusner LL, Kaminski HJ (2015). Editorial: Special issue on standardization of preclinical evaluation of animal models for myasthenia gravis. Exp Neurol.

[3] Smith VM (2021). A functional human-on-a-chip autoimmune disease model of myasthenia gravis for development of therapeutics. Front Cell Dev Biol.

[4] Plomp JJ (2022). A bioassay for neuromuscular junction-restricted complement activation by myasthenia gravis acetylcholine receptor antibodies. J Neurosci Methods.

[5] Hoch W (2001). Auto-antibodies to the receptor tyrosine kinase MuSK in patients with myasthenia gravis without acetylcholine receptor antibodies. Nat Med.

[6] Pham MC (2023). Individual myasthenia gravis autoantibody clones can efficiently mediate multiple mechanisms of pathology. Acta Neuropathol.

[7] Obaid AH (2022). Heterogeneity of acetylcholine receptor autoantibody-mediated complement activity in patients with myasthenia gravis. Neurol Neuroimmunol Neuroinflamm.

[8] Conti-Fine BM (2006). Myasthenia gravis: past, present, and future. J Clin Invest.

[9] Vincent A (2020). Antibodies and receptors: From neuromuscular junction to central nervous system. Neuroscience.

[11] Tran C (2018). Fatigue is a relevant outcome in patients with myasthenia gravis. Muscle Nerve.

[12] Narayanaswami P (2022). Identifying a patient-centered outcome measure for a comparative effectiveness treatment trial in myasthenia gravis. Muscle Nerve.

[13] Regnault A (2023). Measuring overall severity of Myasthenia Gravis (MG): Evidence for the added value of the MG symptoms PRO. Neurol Ther.

[14] Ruiter AM (2021). Prevalence and associated factors of fatigue in autoimmune myasthenia gravis. Neuromuscul Disord.

[15] Kluger BM (2013). Fatigue and fatigability in neurologic illnesses: proposal for a unified taxonomy. Neurology.

[16] Andrapalliyal N (2023). Incidence and causes of overdiagnosis of myasthenia gravis. Muscle Nerve.

[17] Li Z (2023). A multicentre, prospective, double-blind study comparing the accuracy of autoantibody diagnostic assays in myasthenia gravis: the SCREAM study. Lancet Reg Health West Pac.

[18] Gastaldi M (2021). Improving laboratory diagnostics in myasthenia gravis. Expert Rev Mol Diagn.

[19] Kwon YN (2023). Clinical pitfalls and serological diagnostics of MuSK myasthenia gravis. J Neurol.

[20] Klein CJ (2022). LRP4-IgG service line testing in seronegative myasthenia gravis and controls. J Neuroimmunol.

[21] Tzartos JS (2014). LRP4 antibodies in serum and CSF from amyotrophic lateral sclerosis patients. Ann Clin Transl Neurol.

[22] Zisimopoulou P (2014). A comprehensive analysis of the epidemiology and clinical characteristics of anti-LRP4 in myasthenia gravis. J Autoimmun.

[23] Keene KR (2024). Test-retest reliability of repetitive ocular vestibular evoked myogenic potentials in myasthenia gravis patients and healthy control subjects. J Clin Neurophysiol.

[24] Keene KR (2023). Eye muscle MRI in myasthenia gravis and other neuromuscular disorders. J Neuromuscul Dis.

[25] Slater CR (2015). The functional organization of motor nerve terminals. Prog Neurobiol.

[26] Li L (2018). Neuromuscular junction formation, aging, and disorders. Annu Rev Physiol.

[27] Rose N (2022). Receptor clustering and pathogenic complement activation in myasthenia gravis depend on synergy between antibodies with multiple subunit specificities. Acta Neuropathol.

[28] Sanders DB (2014). Does change in acetylcholine receptor antibody level correlate with clinical change in myasthenia gravis?. Muscle Nerve.

[29] Luo J (2010). Specific immunotherapy of experimental myasthenia gravis by a novel mechanism. Ann Neurol.

[30] Albazli K (2020). Complement inhibitor therapy for myasthenia gravis. Front Immunol.

[31] Zhou Y (2007). Anti-C5 antibody treatment ameliorates weakness in experimentally acquired myasthenia gravis. J Immunol.

[32] Vu T (2022). Terminal complement inhibitor ravulizumab in generalized myasthenia gravis. NEJM Evid.

[33] Howard JF (2017). Safety and efficacy of eculizumab in anti-acetylcholine receptor antibody-positive refractory generalised myasthenia gravis (REGAIN): a phase 3, randomised, double-blind, placebo-controlled, multicentre study. Lancet Neurol.

[34] Howard JF (2023). Safety and efficacy of zilucoplan in patients with generalised myasthenia gravis (RAISE): a randomised, double-blind, placebo-controlled, phase 3 study. Lancet Neurol.

[35] Kusner LL (2014). Targeting therapy to the neuromuscular junction: proof of concept. Muscle Nerve.

[36] Lindstrom J, Einarson B (1979). Antigenic modulation and receptor loss in experimental autoimmune myasthenia gravis. Muscle Nerve.

[37] Drachman DB (1978). Myasthenic antibodies cross-link acetylcholine receptors to accelerate degradation. N Engl J Med.

[38] Conti-Tronconi B (1981). Monoclonal antibodies as probes of acetylcholine receptor structure. 2. Binding to native receptor. Biochemistry.

[39] Gomez CM, Richman DP (1983). Anti-acetylcholine receptor antibodies directed against the alpha-bungarotoxin binding site induce a unique form of experimental myasthenia. Proc Natl Acad Sci U S A.

[40] Cetin H (2020). Myasthenia gravis AChR antibodies inhibit function of rapsyn-clustered AChRs. J Neurol Neurosurg Psychiatry.

[41] Vergoossen DLE (2021). Functional monovalency amplifies the pathogenicity of anti-MuSK IgG4 in myasthenia gravis. Proc Natl Acad Sci U S A.

[42] Vergoossen DLE (2020). MuSK antibodies, lessons learned from poly- and monoclonality. J Autoimmun.

[43] Cao M (2020). Myasthenia gravis with antibodies against muscle specific kinase: An update on clinical features, pathophysiology and treatment. Front Mol Neurosci.

[44] Boldingh MI (2015). Geographical distribution of myasthenia gravis in Northern Europe--Results from a population-based study from two countries. Neuroepidemiology.

[45] Rodolico C (2020). MuSK-associated myasthenia gravis: Clinical features and management. Front Neurol.

[46] Boldingh MI (2017). Prevalence and clinical aspects of immigrants with myasthenia gravis in northern Europe. Muscle Nerve.

[47] Hong Y (2018). HLA and MuSK-positive myasthenia gravis: A systemic review and meta-analysis. Acta Neurol Scand.

[48] Yoshikawa H (2022). Two-step nationwide epidemiological survey of myasthenia gravis in Japan 2018. PLoS One.

[49] Dresser L (2021). Myasthenia gravis: Epidemiology, pathophysiology and clinical manifestations. J Clin Med.

[50] Punga AR (2011). MuSK levels differ between adult skeletal muscles and influence postsynaptic plasticity. Eur J Neurosci.

[51] Rodriguez Cruz PM (2015). Clinical features and diagnostic usefulness of antibodies to clustered acetylcholine receptors in the diagnosis of seronegative myasthenia gravis. JAMA Neurol.

[52] Yu Z (2021). Characterization of LRP4/agrin antibodies from a patient with myasthenia gravis. Neurology.

[53] Huijbers MG (2022). Advances in the understanding of disease mechanisms of autoimmune neuromuscular junction disorders. Lancet Neurol.

[54] Weatherbee SD (2006). LDL-receptor-related protein 4 is crucial for formation of the neuromuscular junction. Development.

[55] Kim N (2008). Lrp4 is a receptor for agrin and forms a complex with MuSK. Cell.

[56] Zhang B (2008). LRP4 serves as a coreceptor of agrin. Neuron.

[57] Chuquisana O (2024). Functional signature of LRP4 antibodies in myasthenia gravis. Neurol Neuroimmunol Neuroinflamm.

[58] Lazaridis K, Tzartos SJ (2020). Autoantibody specificities in myasthenia gravis; Implications for improved diagnostics and therapeutics. Front Immunol.

[59] Zhang B (2014). Autoantibodies to agrin in myasthenia gravis patients. PLoS One.

[60] Wang S (2021). Antibodies to full-length agrin protein in Chinese patients with myasthenia gravis. Front Immunol.

[61] Gilhus NE, Verschuuren JJ (2015). Myasthenia gravis: subgroup classification and therapeutic strategies. Lancet Neurol.

[62] Lee JY (2016). Compromised fidelity of B-cell tolerance checkpoints in AChR and MuSK myasthenia gravis. Ann Clin Transl Neurol.

[63] Verdier J (2023). Single-cell mass cytometry on peripheral cells in myasthenia gravis identifies dysregulation of innate immune cells. Front Immunol.

[64] Ingelfinger F (2021). Single-cell profiling of myasthenia gravis identifies a pathogenic T cell signature. Acta Neuropathol.

[65] Cron MA (2020). Role of miRNAs in normal and myasthenia gravis thymus. Front Immunol.

[66] Sengupta M (2014). Serum metabolomic response of myasthenia gravis patients to chronic prednisone treatment. PLoS One.

[67] Payet CA (2022). Myasthenia gravis: An acquired interferonopathy?. Cells.

[68] Molin CJ (2018). Thymectomy lowers the myasthenia gravis biomarker miR-150-5p. Neurol Neuroimmunol Neuroinflamm.

[69] Marx A (2012). Thymus pathology observed in the MGTX trial. Ann N Y Acad Sci.

[70] Cufi P (2014). Central role of interferon-beta in thymic events leading to myasthenia gravis. J Autoimmun.

[71] Meraouna A (2006). The chemokine CXCL13 is a key molecule in autoimmune myasthenia gravis. Blood.

[72] Berrih-Aknin S (2009). CCL21 overexpressed on lymphatic vessels drives thymic hyperplasia in myasthenia. Ann Neurol.

[73] Leite MI (2007). Myasthenia gravis thymus: complement vulnerability of epithelial and myoid cells, complement attack on them, and correlations with autoantibody status. Am J Pathol.

[74] Payet CA (2023). Central role of macrophages and nucleic acid release in myasthenia gravis thymus. Ann Neurol.

[75] Vanderlugt CL (1998). The functional significance of epitope spreading and its regulation by co-stimulatory molecules. Immunol Rev.

[76] Newsom-Davis J (1981). Thymus cells in myasthenia gravis selectively enhance production of anti-acetylcholine-receptor antibody by autologous blood lymphocytes. N Engl J Med.

[77] Jiang R (2020). Thymus-derived B cell clones persist in the circulation after thymectomy in myasthenia gravis. Proc Natl Acad Sci U S A.

[78] Uzawa A (2021). Roles of cytokines and T cells in the pathogenesis of myasthenia gravis. Clin Exp Immunol.

[79] Ramanujam R (2011). Utilizing twins concordance rates to infer the predisposition to myasthenia gravis. Twin Res Hum Genet.

[80] Green JD (2020). Epidemiological evidence for a hereditary contribution to myasthenia gravis: a retrospective cohort study of patients from North America. BMJ Open.

[81] Saruhan-Direskeneli G (2016). Genetic heterogeneity within the HLA region in three distinct clinical subgroups of myasthenia gravis. Clin Immunol.

[82] Avidan N (2014). Genetic basis of myasthenia gravis - a comprehensive review. J Autoimmun.

[83] Handunnetthi L (2021). Genomic insights into myasthenia gravis identify distinct immunological mechanisms in early and late onset disease. Ann Neurol.

[84] Kakalacheva K (2011). Intrathymic Epstein-Barr virus infection is not a prominent feature of myasthenia gravis. Ann Neurol.

[85] Cavalcante P (2011). Inflammation and Epstein-Barr virus infection are common features of myasthenia gravis thymus: possible roles in pathogenesis. Autoimmune Dis.

[86] Leopardi V (2021). A systematic review of the potential implication of infectious agents in myasthenia gravis. Front Neurol.

[87] Conrad N (2023). Incidence, prevalence, and co-occurrence of autoimmune disorders over time and by age, sex, and socioeconomic status: a population-based cohort study of 22 million individuals in the UK. Lancet.

[88] Goronzy JJ, Weyand CM (2012). Immune aging and autoimmunity. Cell Mol Life Sci.

[89] Chia R (2022). Identification of genetic risk loci and prioritization of genes and pathways for myasthenia gravis: a genome-wide association study. Proc Natl Acad Sci U S A.

[90] Seldin MF (2016). Genome-wide association study of late-onset myasthenia gravis: Confirmation of TNFRSF11A and identification of ZBTB10 and three distinct HLA associations. Mol Med.

[91] Wolfe GI (2016). Randomized trial of thymectomy in myasthenia gravis. N Engl J Med.

[92] Yamada Y (2020). Thymoma associated myasthenia gravis (TAMG): Differential expression of functional pathways in relation to MG status in different thymoma histotypes. Front Immunol.

[93] Marx A (2015). Thymoma related myasthenia gravis in humans and potential animal models. Exp Neurol.

[94] Buckley C (2001). Mature, long-lived CD4+ and CD8+ T cells are generated by the thymoma in myasthenia gravis. Ann Neurol.

[95] Strobel P (2004). Selective loss of regulatory T cells in thymomas. Ann Neurol.

[96] Zheng K (2012). PTPN22 and CTLA-4 gene polymorphisms in resected thymomas and thymus for myasthenia gravis. Thorac Cancer.

[97] Chuang WY (2009). The PTPN22gain-of-function+1858T(+) genotypes correlate with low IL-2 expression in thymomas and predispose to myasthenia gravis. Genes Immun.

[98] Yasumizu Y (2022). Myasthenia gravis-specific aberrant neuromuscular gene expression by medullary thymic epithelial cells in thymoma. Nat Commun.

[99] Martins F (2019). Adverse effects of immune-checkpoint inhibitors: epidemiology, management and surveillance. Nat Rev Clin Oncol.

[100] Sullivan RJ, Weber JS (2022). Immune-related toxicities of checkpoint inhibitors: mechanisms and mitigation strategies. Nat Rev Drug Discov.

[101] Safa H (2019). Immune checkpoint inhibitor related myasthenia gravis: single center experience and systematic review of the literature. J Immunother Cancer.

[102] Yu W (2015). Clonal deletion prunes but does not eliminate self-specific αβ CD8(+) T lymphocytes. Immunity.

[103] Allen S (2011). Shaping the T-cell repertoire in the periphery. Immunol Cell Biol.

[104] Hendricks TM (2019). Incidence, epidemiology, and transformation of ocular myasthenia gravis: A population-based study. Am J Ophthalmol.

[105] Nagaishi A (2021). Autoantibodies in Japanese patients with ocular myasthenia gravis. Muscle Nerve.

[106] Jacob S (2012). Presence and pathogenic relevance of antibodies to clustered acetylcholine receptor in ocular and generalized myasthenia gravis. Arch Neurol.

[107] Wang ZY (2000). T cell recognition of muscle acetylcholine receptor in ocular myasthenia gravis. J Neuroimmunol.

[108] Wilson L, Davis H (2023). The role of thymoma and thymic hyperplasia as prognostic risk factors for secondary generalisation in adults with ocular myasthenia gravis: A systematic narrative review. Br Ir Orthopt J.

[109] Matsui N (2009). Increasing incidence of elderly onset patients with myasthenia gravis in a local area of Japan. J Neurol Neurosurg Psychiatry.

[110] Grob D (2008). Lifetime course of myasthenia gravis. Muscle Nerve.

[111] Zhang X (2007). Clinical and serological study of myasthenia gravis in HuBei Province, China. J Neurol Neurosurg Psychiatry.

[112] Zhu WH (2012). HLA-DQA1*03:02/DQB1*03:03:02 is strongly associated with susceptibility to childhood-onset ocular myasthenia gravis in Southern Han Chinese. J Neuroimmunol.

[113] Wang J (2023). Environmental factors affecting the risk of generalization for ocular-onset myasthenia gravis: a nationwide cohort study. QJM.

[114] Sabre L (2019). miR-30e-5p as predictor of generalization in ocular myasthenia gravis. Ann Clin Transl Neurol.

[115] Huijbers MG (2013). MuSK IgG4 autoantibodies cause myasthenia gravis by inhibiting binding between MuSK and Lrp4. Proc Natl Acad Sci U S A.

[116] Leite MI (2005). Fewer thymic changes in MuSK antibody-positive than in MuSK antibody-negative MG. Ann Neurol.

[117] Clifford KM (2019). Thymectomy may not be associated with clinical improvement in MuSK myasthenia gravis. Muscle Nerve.

[118] Hehir MK (2017). Rituximab as treatment for anti-MuSK myasthenia gravis: Multicenter blinded prospective review. Neurology.

[119] Stathopoulos P (2017). Autoantibody-producing plasmablasts after B cell depletion identified in muscle-specific kinase myasthenia gravis. JCI Insight.

[120] Kooshesh KA (2023). Health consequences of thymus removal in adults. N Engl J Med.

[121] Kaminski HJ (2024). Does surgical removal of the thymus have deleterious consequences?. Neurology.

[122] Narayanaswami P (2021). International consensus guidance for management of myasthenia gravis: 2020 Update. Neurology.

[123] Murai M (2023). The Japanese clinical guidelines 2022 for myasthenia gravis and Lambert–Eaton myasthenic syndrome. Clin Exp Neuroimmunol.

[124] Sussman J (2018). The Association of British Neurologists’ myasthenia gravis guidelines. Ann N Y Acad Sci.

[125] Kaminski HJ, Denk J (2022). Corticosteroid treatment-resistance in myasthenia gravis. Front Neurol.

[126] Antonini G (2023). Real world study on prevalence, treatment and economic burden of myasthenia gravis in Italy. Heliyon.

[127] Shen SP (2023). Healthcare resource utilization and costs associated with generalized myasthenia gravis: a retrospective matched cohort study using the National Health Insurance Research Database in Taiwan. Front Neurol.

[128] Howard JF (2021). Safety, efficacy, and tolerability of efgartigimod in patients with generalised myasthenia gravis (ADAPT): a multicentre, randomised, placebo-controlled, phase 3 trial. Lancet Neurol.

[129] Bril V (2023). Safety and efficacy of rozanolixizumab in patients with generalised myasthenia gravis (MycarinG): a randomised, double-blind, placebo-controlled, adaptive phase 3 study. Lancet Neurol.

[130] Ward ES, Ober RJ (2018). Targeting FcRn to generate antibody-based therapeutics. Trends Pharmacol Sci.

[131] Nelke C (2022). Neonatal Fc receptor-targeted therapies in neurology. Neurotherapeutics.

[132] Gomez CM (1984). Induction of the morphologic changes of both acute and chronic experimental myasthenia by monoclonal antibody directed against acetylcholine receptor. Acta Neuropathol.

[133] Engel AG (1976). The motor end plate in myasthenia gravis and in experimental autoimmune myasthenia gravis. A quantitative ultrastructural study. Ann N Y Acad Sci.

[134] Menon D, Bril V (2022). Pharmacotherapy of generalized myasthenia gravis with special emphasis on newer biologicals. Drugs.

[135] Rother RP (2007). Discovery and development of the complement inhibitor eculizumab for the treatment of paroxysmal nocturnal hemoglobinuria. Nat Biotechnol.

[136] Tang GQ (2023). Zilucoplan, a macrocyclic peptide inhibitor of human complement component 5, uses a dual mode of action to prevent terminal complement pathway activation. Front Immunol.

[137] Nichols J, Eppes S (2022). Meningococcal vaccination: an update on meningococcal vaccine recommendations for the primary care physician. Dela J Public Health.

[138] Kusner LL (2019). Investigational RNAi therapeutic targeting C5 is efficacious in pre-clinical models of myasthenia gravis. Mol Ther Methods Clin Dev.

[139] Kuboi Y (2023). Identification of potent siRNA targeting complement C5 and its robust activity in pre-clinical models of myasthenia gravis and collagen-induced arthritis. Mol Ther Nucleic Acids.

[140] Piehl F (2022). Efficacy and safety of rituximab for new-onset generalized myasthenia gravis: The RINOMAX Randomized Clinical Trial. JAMA Neurol.

[141] Alabbad S (2020). Monoclonal antibody-based therapies for myasthenia gravis. BioDrugs.

[142] Rejdak K (2020). Cladribine in myasthenia gravis: a pilot open-label study. Eur J Neurol.

[143] Dhillon S (2021). Telitacicept: first approval. Drugs.

[144] Gomez AM (2014). Proteasome inhibition with bortezomib depletes plasma cells and specific autoantibody production in primary thymic cell cultures from early-onset myasthenia gravis patients. J Immunol.

[145] Kohler S (2019). Bortezomib in antibody-mediated autoimmune diseases (TAVAB): study protocol for a unicentric, non-randomised, non-placebo controlled trial. BMJ Open.

[146] Granit V (2023). Safety and clinical activity of autologous RNA chimeric antigen receptor T-cell therapy in myasthenia gravis (MG-001): a prospective, multicentre, open-label, non-randomised phase 1b/2a study. Lancet Neurol.

[147] Monfardini C (2002). Adoptive protection from experimental myasthenia gravis with T cells from mice treated nasally with acetylcholine receptor epitopes. J Neuroimmunol.

[148] Weathington NM, Blalock JE (2003). Rational design of peptide vaccines for autoimmune disease: harnessing molecular recognition to fix a broken network. Expert Rev Vaccines.

[149] Luo J, Lindstrom J (2015). AChR-specific immunosuppressive therapy of myasthenia gravis. Biochem Pharmacol.

[150] Deng H (2019). Associations of BAFF rs2893321 polymorphisms with myasthenia gravis susceptibility. BMC Med Genet.

[151] Hewett K (2018). Randomized study of adjunctive belimumab in participants with generalized myasthenia gravis. Neurology.

[152] GomezMancilla B (2024). Efficacy and safety of iscalimab, a novel anti-CD40 monoclonal antibody, in moderate-to-severe myasthenia gravis: a phase 2 randomized study. J Clin Neurosci.

[153] Sikorski P (2023). Serum metabolomics of treatment response in myasthenia gravis. PLoS One.

[154] Xie Y (2016). GR gene polymorphism is associated with inter-subject variability in response to glucocorticoids in patients with myasthenia gravis. Eur J Neurol.

[155] Xie Y (2017). The role of osteopontin and its gene on glucocorticoid response in myasthenia gravis. Front Neurol.

